# Expression changes of cytotoxicity and apoptosis genes in HTLV-1-associated myelopathy/tropical spastic paraparesis patients from the perspective of system virology

**DOI:** 10.1099/acmi.0.000088

**Published:** 2020-01-14

**Authors:** Kiarash Ghazvini, Masoud Youssefi, Masoud Keikha

**Affiliations:** ^1^​ Antimicrobial Resistance Research Center, Mashhad University of Medical Sciences, Mashhad, Iran; ^2^​ Department of Microbiology and Virology, Faculty of Medicine, Mashhad University of Medical Sciences, Mashhad, Iran

**Keywords:** apoptosis, cytotoxicity, HAM/TSP, HTLV-1, pathogenesis

## Abstract

Although human T-cell lymphotropic virus type-1 (HTLV-1) was the first retrovirus among human pathogens to be identified, insufficient information on the pathogenesis of HTLV-1 infection means that no precise mechanism has yet been provided for HTLV-1-associated myelopathy/tropical spastic paraparesis (HAM/TSP). Based on previous studies, it was found that apoptosis and inflammation stimulation were among the most important mechanisms underlying HAM/TSP. The present study provides an *in-silico* analysis of the microarray data related to HAM/TSP patients. Expression changes of the genes responsible for cytotoxicity and apoptosis processes of HAM/TSP patients and asymptomatic carriers were investigated. Expression of the genes involved in cytotoxicity and apoptosis in HAM/TSP patients was decreased; hence, a model was proposed indicating that the spread of immune responses in HAM/TSP may be due to expression of HTLV-1 virulence factors and the resistance of HTLV-1-infected cells to apoptosis.

## Introduction

Human T-cell lymphotropic virus type-1 (HTLV-1) is one of the most important members of the family *Retroviridae* and was first isolated from a young patient with cutaneous T-cell lymphoma by Robert Gallo *et al*. in 1980 [1]. HTLV-1 can cause various infections such as adult T-cell leukaemia lymphoma (ATLL), HTLV-1-associated myelopathy/tropical spastic paraparesis (HAM/TSP), uveitis, arthritis, lymphadenitis, infective dermatitis and alveolitis [2]. Based on the available evidence, 15–20 million people are infected with the virus worldwide, most of whom remain asymptomatic carriers (ACs), while 2–6 % have ATLL and 2–3 % have HAM/TSP [1–3]. No precise mechanism is provided for this phenomenon. However, expression of viral virulence factors such as *Tax* and HTLV-1 bZIP factor (*HBZ*), and host epigenetic and immune changes, in particular expression of cytokines, activate NK cells and T-lymphocytes; the altered cell signalling pathways subsequently promote progress toward HAM/TSP and ATLL [3–5].

The immune system employs a variety of approaches including apoptosis of infected cells, production of granzymes and perforin enzymes, release of nitric oxide radicals, phagocytosis, and antibody production to eliminate infectious agents, especially HTLV-1 [5]. However, HTLV-1 is one of the most exclusive pathogen viruses of humans, and reduces the immune response by infecting immune cells especially CD4+ T cells [5–7]. HAM/TSP is a chronic neurodegenerative inflammation. It appears that cytotoxic T lymphocytes (CTLs) and CD4+ T cells accumulate in the spinal cord locus during the immunopathogenesis in response to chemokines CXC10, CXCL9, TNF-α and CCL2 produced by the infected astrocytes during HTLV-1 infection, causing inflammation and destruction of neurons [1, 8].

Previous studies have shown that HTLV-1-infected cells undergo apoptosis, which is an important mechanism for virus elimination [5, 9]. However, the mechanism of pathogenesis of HTLV-1 is still not properly known. Studying the pathogenesis pathways of this virus at the transcriptomic level will lead to a better understanding of its pathogenesis and developing diagnosic and treatment methods of HTLV-1 infections [9, 10]. The purpose of this study was to investigate the expression changes of genes associated with planned survival and cell death processes using microarray data. Moreover, we evaluated *Fas, FasL*, perforin, granzyme, granulysin, *bax, Bad*, BH3-family, *BCL2* and caspase8 gene expression changes between the AC and HAM/TSP groups, and a molecular signalling network is presented for the pathogenesis of HTLV-1 based on the target genes.

## Method

The gene expression profiles of polymorphonuclear neutrophils were primarily obtained in ACs and HAM/TSP individuals under accession number GSE19080 from the NCBI Gene Expression Omnibus (GEO) database. Microarray data were then obtained individually from the AC and HAM/TSP groups. In addition, the GEO2R software and R.3.2.5 software (MetaQC and Affy packages) were used to calculate the fold change and expression changes of differentially expressed genes (DEGs) between the AC and HAM/TSP groups. Analysis of DEGs was performed using Benjamini–Hochberg false discovery rate (FDR)-adjusted *P*-values ​​<0.05, and positive fold change numbers were used to indicate upregulation and the negative fold change values ​​downregulation [9]. A heatmap plot was also created using the online server www.hiv.lanl.gov based on the default instructions.

The Protein–Protein Interaction Network (PPIN) was plotted using the online STRING (Search Tool for the Retrieval of Interacting Genes) server, and the KEGG and Enrichr (Wikipathway, GO Biological process and Panther services) databases were used for gene ontology. Finally, the molecular signalling network was determined according to the collected information.

## Results

**Fig. 1.**
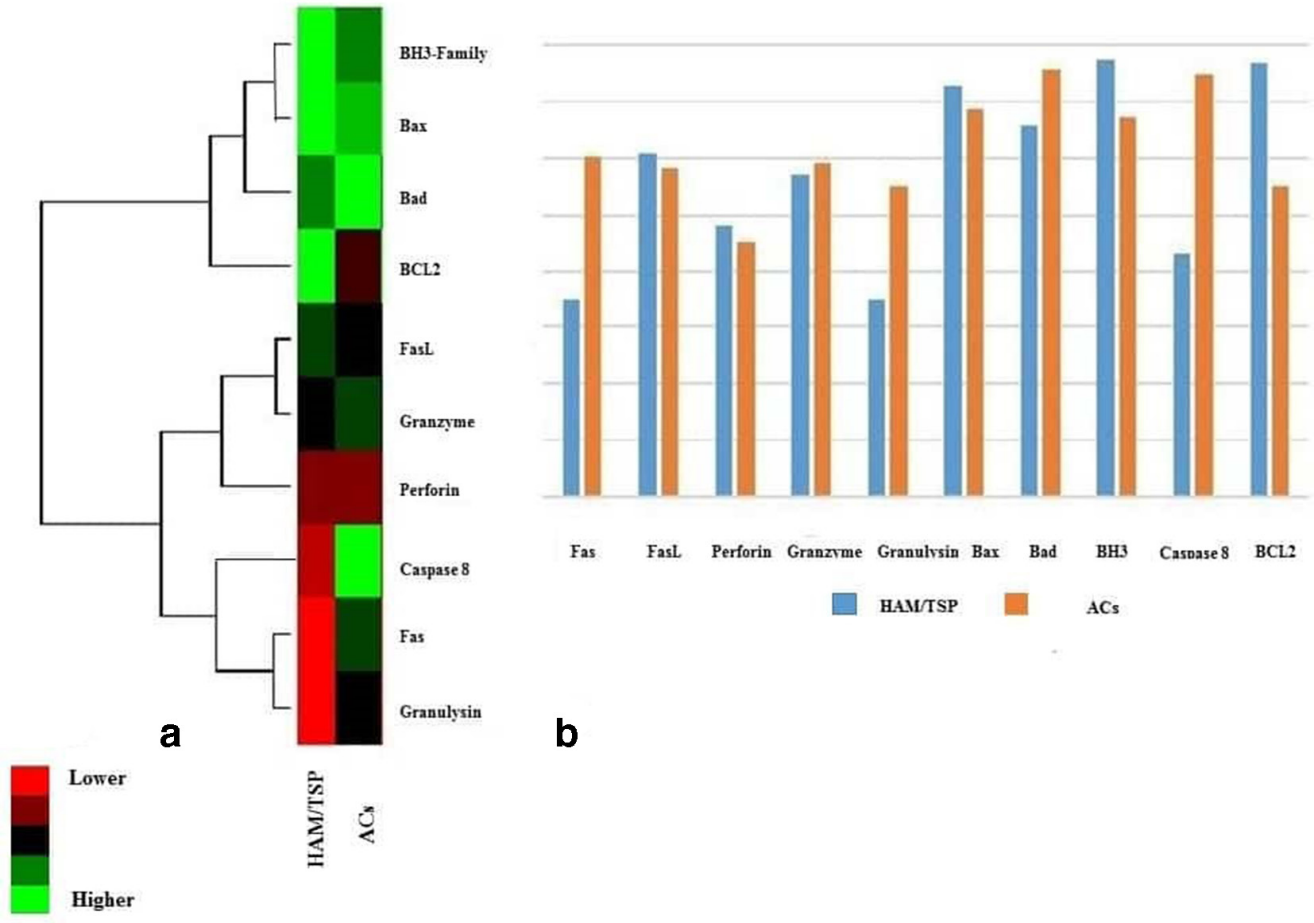
Expression profiles of cytotoxicity- and apoptosis-related genes between ACs and HAM/TSP patients. (a) Heatmap of hub genes between ACs and HAM/TSP patients in which colours demonstrate the expression level of each gene; (b) diagram of upregulated and downregulated hub genes between ACs (orange) and HAM/TSP patients (blue).

According to our study aims, the expression changes of the most important genes involved in the apoptosis process, including Fas, FasL, perforin, granzyme, granulysin, Bax, Bad, BH3-family, Caspase8 and BCL2, were investigated in ACs and HAM/TSP patients (Fig. 1 and Table 1). In general, the expression of inducer genes initiating apoptosis in HAM/TSP patients was lower than that in the AC population. Moreover, the expression of cytotoxic genes such as granzyme and granulysin was decreased in the HAM/TSP group. Although the expression of all the cellular cytotoxicity and apoptosis genes in HAM/TSP patients was decreased, only the expression of Fas and Caspase 8 genes was significantly different between the AC and HAM/TSP groups.

**Table 1.** List of upregulated (positive logFC) and downregulated (negative logFC) hub genes in HAM/TSP patients vs. ACs FC, fold change.

Gene	logFC
Fas	−1.29
FasL	0.61
Perforin	0.19
Granzyme	−0.17
Granulysin	−1.48
Bax	0.61
Bad	−0.79
BH3-family	0.38
Caspase 8	−1.11
BCL2	−1.1

**Fig. 2.**
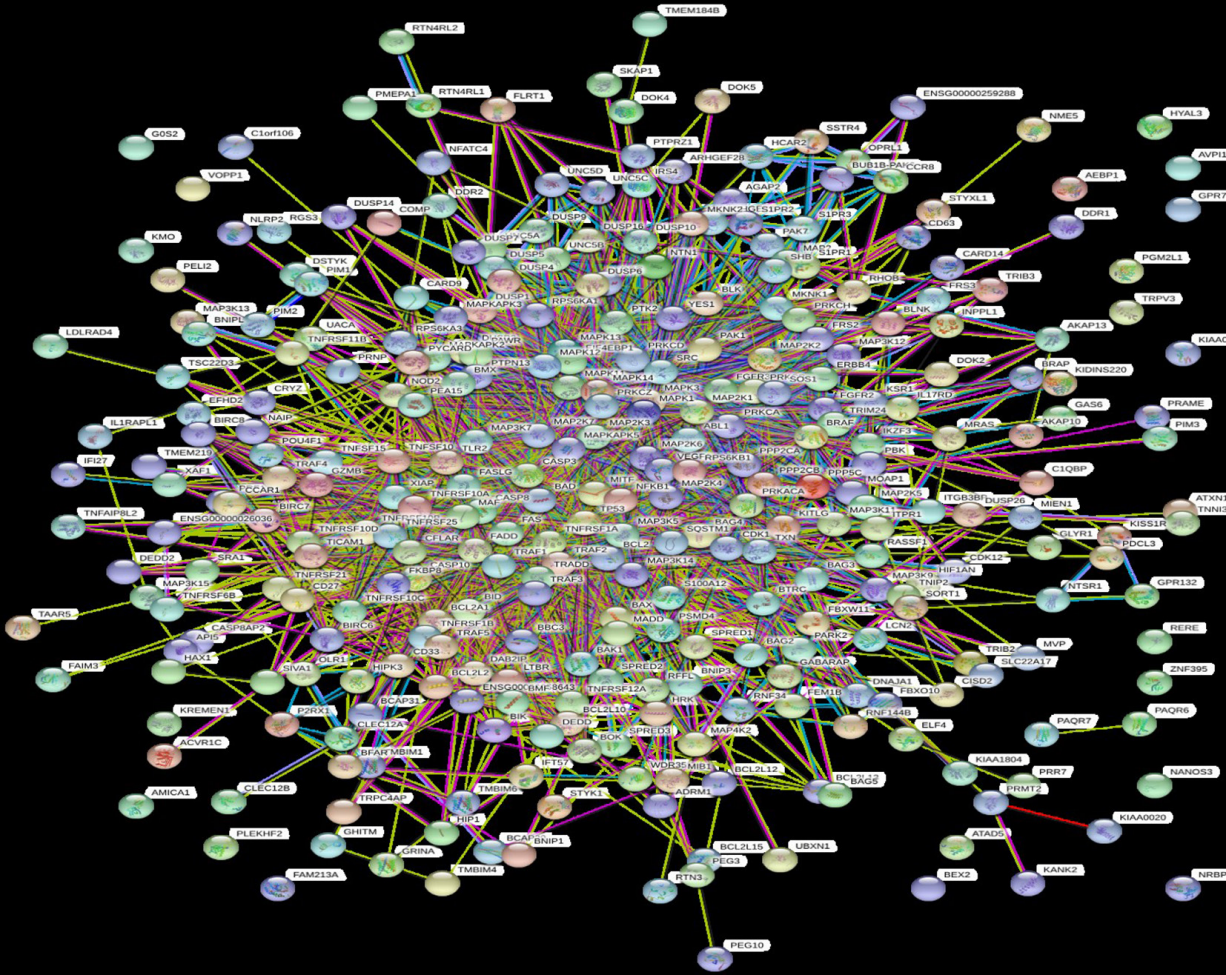
Protein–protein interaction network system between hub genes and closely related genes during the HAM/TSP state. The distance between each node represents the interaction probability, and each node consisting of a coloured, white and empty node represents a query, closely related-query and unknown protein, respectively.

Analyses of the gene network indicated that genes associated with the process of apoptosis and the cell cytoskeleton are surrounded by genes in the immune system, and the differentiation and survival process is dependent on immune system changes. Furthermore, the genes responsible for the PI3K-Akt, JNK, NF_κB, TP53 and MAPK signalling pathways are the most common genes that form the gene network (Fig. 2). According to this network, there are close relationships between the genes responsible for the process of programmed cell death, transcription factors, the TP53 signalling pathway and mitogen-activated protein kinases (MAPKs), whereas those responsible for cytoskeleton cellular rearrangement, autophagy and cytokine production are further away from the central nodes.

**Fig. 3.**
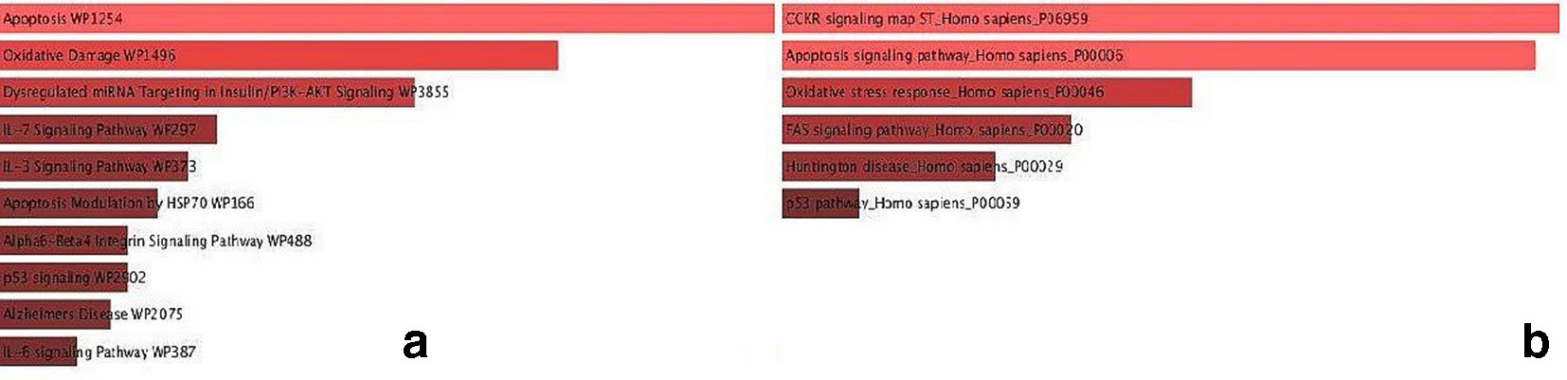
The most enriched pathways of the hub genes based on gene ontology analysis using enricher databases. (a) WikiPathway 2019; according to this server, most hub genes are involved in apoptosis, oxidative stress, PI3K/Akt-mTOR, pro-inflammatory interleukins as well as the cell survival signalling pathway. (b) Biological process; this indicates that the processed hub genes are from the dysregulating apoptosis, oxidative stress, FasL and p53 signalling pathways that play key roles in death, cell survival and central nervous system (CNS) neurodegeneration.

According to the gene ontology data, the analysed genes regulate various processes including apoptosis, oxidative stress, TP53 signalling, and the production of pro-inflammatory cytokines such as IL-6, 3 and 7 (Fig. 3). Based on the gene ontology data, HAM/TSP phenomena such as inflammation, tissue invasion, tissue destruction and cell survival are activated by various proteins (e.g. PI3K, p53, MAPK, NF_κB and IRF1). Moreover, following increased activity, the signalling pathways may lead to immune system deficiencies or autoimmune diseases.

**Fig. 4.**
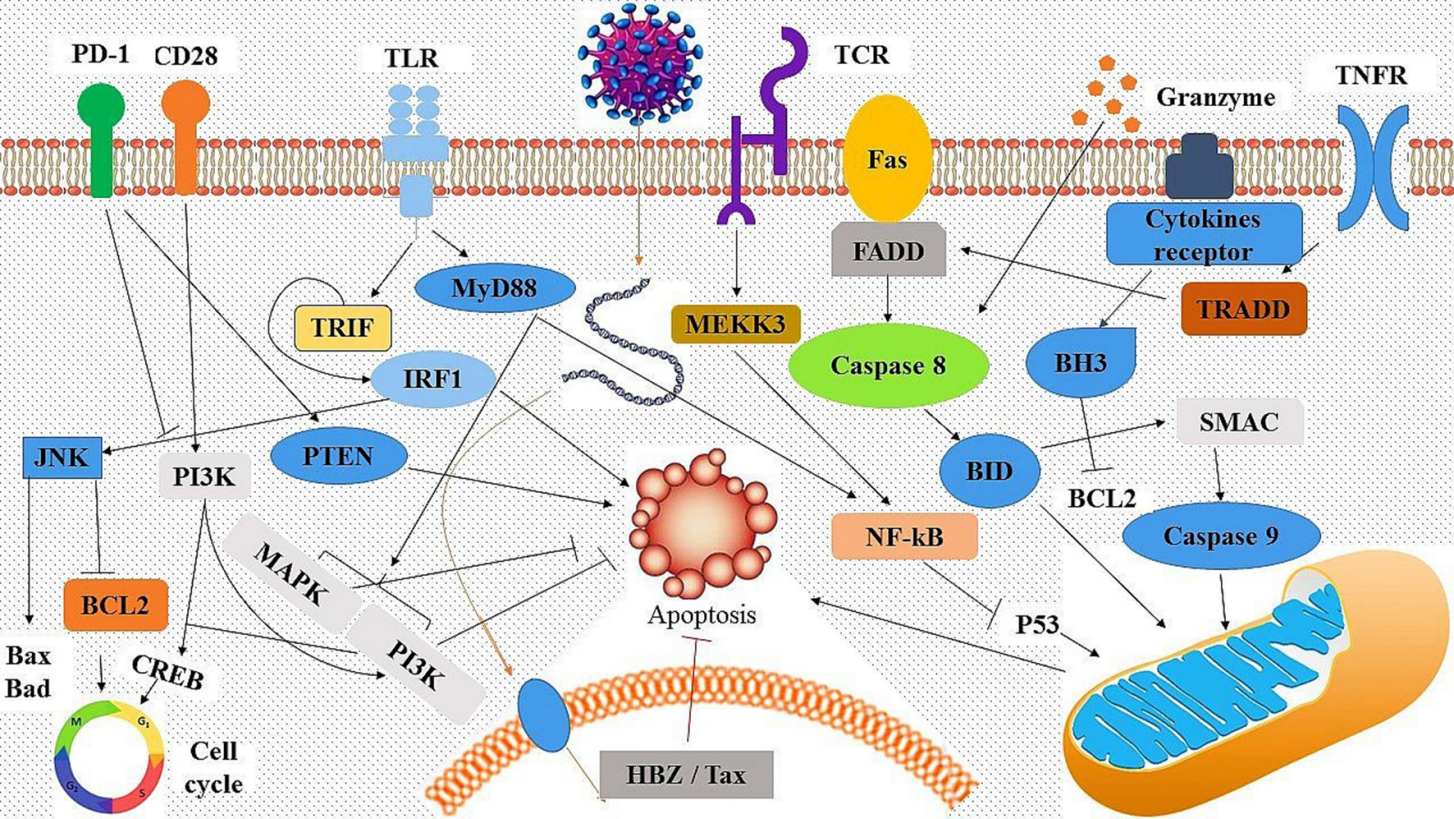
The proposed signalling network which occurs during HAM/TSP based on the hub genes. Induction with death receptors (i.e. Fas, TNFR and PD-1) causes apoptosis and cytotoxic enzymes, and activation of IRF1 leads to activation of caspase cascade and cell death. By contrast, CD28, TCR, and the cytokine receptor signal network influence PI3K-Akt, mTOR, NF_κB, CREB, BH3-Only family activation as well as inhibition of cyclin-dependent kinases, which promote cell survival and T cell proliferation. In addition, HTLV-1 virulence factors, particularly *Tax* and *HBZ*, can support T cell proliferation using various signalling pathways which prevent apoptosis activation.

Finally, the molecular signalling network was presented based on the findings (Fig. 4). It appears that the decrease in apoptosis in HAM/TSP patients compared to ACs may be due to the continued stimulation of HTLV-1-induced cell proliferation, migration of polymorphonuclear neutrophils (PMNs) to the spinal cord, and expression of HTLV-1 virulence factors such as *Tax* and *HBZ*. In general, it appears that the decrease in apoptosis and cytotoxicity in HAM/TSP patients is due to the expression of HTLV-1 virulence factors and the continuous stimulation of PMN migration by chemokines produced by astrocytes, neurons and oligodendrocytes.

## Discussion

Despite extensive studies of the immunopathogenesis of HTLV-1, no precise mechanism has yet been provided for its links to ATLL and HAM/TSP. On the other hand, as the host epigenetic changes also play an important role in the progression of infection to the disease, extensive studies at the transcriptomic level could provide important information on the pathogenesis of HTLV-1 and the mechanism leading to ATLL and HAM/TSP [11, 12]. In addition, by identifying gene mutations, mRNA as well as protein expression changes, the microarray assay can be considered an important tool for immunopathogenesis, diagnostic and therapeutic studies of HTLV-1 infection [9, 13].

HAM/TSP is a chronic neuro-inflammatory infection, which develops slowly but persistently, eventually leading to paralysis [14]. Multi-organ lymphocyte infiltration, overexpression of T-bet transcription factor, cytokine IFN-γ production and spontaneous proliferation of lymphocytes are among the most important features of this disease [5, 15, 16]. Based on the available evidence, HTLV-1 initially infects the long-living CD4+CD25+CCR4+ T regulatory cells, and uses these cells as a reservoir. These cells can produce IFN-γ and trigger themselves to progress towards HAM/TSP [17]. Gradually, the IFN-γ produced CD4+CD25+CCR4+-infected T cells stimulate transcription factor T-bet activity and lead to Th1 induction and ultimately produce large amounts of IFN-γ as well as inflammation of the CNS [17, 18]. It has been shown that IFN-γ production in HAM/TSP patients is significantly higher than that in ACs [19, 20]. Moreover, the virulence factors of HTLV-1, especially *Tax*, increase Th1 production and consequently increase the inflammation and tissue destruction in the CNS [21, 22]. Based on previous studies, the rate of *Tax* transcription is much higher in HAM/TSP patients than in ACs and ATLL patients [5, 22].

Cytolytic enzymes and apoptosis are the most important mechanisms of the immune system against viral infections, and do so by destroying cells infected with the virus [23]. Perforin, granzyme and granulysin are the most important cytolytic enzymes that cause cell death via pores in the cytoplasmic membrane of the target cells, disrupting the electrochemical gradient of the cells, and damaging the DNA [24].

Following the stimulation of FasR, an intermediate molecule termed FADD, which can stimulate cellular apoptosis by stimulating caspase 8, is activated; the BH3-family also induces cytochrome C release from the mitochondria and causes the activation of caspase cascade by affecting Bad and Bax, ultimately leading to cell death [25].

In the present study, expression changes of key genes of the cytotoxic and apoptosis pathways between the AC and HAM/TSP groups were compared and evaluated. According to the study results, overall expression of cytolytic enzymes in the HAM/TSP group was partially reduced. In addition, the results showed an apparent reduction in the expression of the genes involved in cell apoptosis in the HAM/TSP group, such that there was a significant difference between the Fas and caspase 8 genes. However, it should be noted that these are preliminary hypotheses and further studies are needed for confirmation. Based on a review of studies, the process of apoptosis is one of the most important approaches for the treatment of HTLV-1 infection, particularly for the elimination of IL-2-dependent CD4+ T cells in ATLL patients [23, 26–28]. According to Mohammadi *et al*., expression of the genes involved in cell cytotoxicity and apoptosis in HAM/TSP patients is lower than in ACs [5]. In another study, the effect of curcumin on the increase in cytotoxicity and apoptosis activity of HTLV-1-infected cells was investigated in HAM/TSP patients and ACs, and it was found that subsequent to consuming curcumin, expression of genes in the apoptosis and cytolytic enzymes significantly increased in AC patients [29]. It has also been demonstrated in several studies that targeting and blocking the TP53, Rb/E2F-1, JAK/STAT and IFN/STAT1/Fas axis pathways can be used as therapeutic alternatives for the treatment and control of HTLV-1 infection by affecting the apoptosis of HTLV-1-infected cells [16–28, 30, 31]. Despite conflicting studies in this regard, according to the study of Mozhgani *et al*., the increased expression of MSH2 (a DNA repair protein) in HAM/TSP patients can lead to increased apoptosis [9].

A study carried out by Mühleisen *et al*. has also shown that overexpression of *Tax* can inhibit Bid and Bim expression and the resistance of HTLV-1-infected cells to apoptosis [32]. However, Ohshima *et al*. found that HTLV-1 *HBZ* leads to a decrease in apoptosis by inhibiting the IRF-1 signalling pathway [33]. Mozhgani *et al*. also suggested that overexpression of PSMB8 increases apoptosis during HTLV-1 infection [13]. However, previous studies have shown that apoptosis plays a critical role in the pathogenesis of HTLV-1 infections and is affected by several signalling pathways such as IRF1, NF_κB, PI3K-Akt, TP53 and MAPKs, and targeting this pathway will be important for the development of new anti-HTLV-1 drugs [25–31].

### Conclusion

Expression of the genes responsible for cytotoxic activities and apoptosis was reduced in HAM/TSP patients compared to ACs, and it appears that the survival of HTLV-1-infected T cells (HTLV-1-infected CD4+ T cells) stimulates and increases IFN-γ production, increases lymphocyte responses and leads to progression towards HAM/TSP. However, this hypothesis cannot be confirmed due to inconsistent literature results, and further studies in this area are needed. Furthermore, based on previous literature reviews, it can be concluded that the processes of cell differentiation, cell survival and apoptosis are among the most important cellular processes that play important roles in the pathogenesis of HTLV-1 infections. Studying and targeting these pathways may lead to the introduction of anti-HTLV-1 drugs.
